# Modulation of the Gal-9/TIM-3 Immune Checkpoint with α-Lactose. Does Anomery of Lactose Matter?

**DOI:** 10.3390/cancers13246365

**Published:** 2021-12-18

**Authors:** Christian Bailly, Xavier Thuru, Bruno Quesnel

**Affiliations:** 1 OncoWitan, Scientific Consulting Office, 59290 Lille, France; 2 University of Lille, CNRS, Inserm, CHU Lille, UMR9020—UMR1277—Canther—Cancer Heterogeneity, Plasticity and Resistance to Therapies, 59000 Lille, France; xavier.thuru@univ-lille.fr (X.T.); bruno.quesnel@chru-lille.fr (B.Q.)

**Keywords:** lactose, galectin-9, PD-1/PD-L1, TIM-3, soluble PD-L1, immune checkpoint, cancer

## Abstract

**Simple Summary:**

The disaccharide lactose is a common excipient in pharmaceutical products. In addition, the two anomers α- and β-lactose can exert immuno-modulatory effects. α-Lactose functions as a major regulator of the T-cell immunoglobulin mucin-3 (Tim-3)/Galectin-9 (Gal-9) immune checkpoint, through direct binding to the β-galactoside-binding lectin galectin-9. The blockade of TIM-3 with monoclonal antibodies or small molecules represents a promising approach to combat onco-hematological diseases, in particular myelodysplastic syndromes, and acute myeloid leukemia. Alternatively, the activity of the checkpoint can be modulated via targeting of Gal-9 with both α- and β-lactose. In fact, lactose is a quasi-pan-galectin ligand, capable of modulating the functions of most of the 16 galectin molecules. This review discusses the capacity of lactose and Gal-9 to modulate the TIM-3/Gal-9 and PD-1/PD-L1 immune checkpoints in oncology. The immuno-regulatory roles of lactose and Gal-9 are highlighted.

**Abstract:**

The disaccharide lactose is an excipient commonly used in pharmaceutical products. The two anomers, α- and β-lactose (α-L/β-L), differ by the orientation of the C-1 hydroxyl group on the glucose unit. In aqueous solution, a mutarotation process leads to an equilibrium of about 40% α-L and 60% β-L at room temperature. Beyond a pharmaceutical excipient in solid products, α-L has immuno-modulatory effects and functions as a major regulator of TIM-3/Gal-9 immune checkpoint, through direct binding to the β-galactoside-binding lectin galectin-9. The blockade of the co-inhibitory checkpoint TIM-3 expressed on T cells with anti-TIM-3 antibodies represents a promising approach to combat different onco-hematological diseases, in particular myelodysplastic syndromes and acute myeloid leukemia. In parallel, the discovery and development of anti-TIM-3 small molecule ligands is emerging, including peptides, RNA aptamers and a few specifically designed heterocyclic molecules. An alternative option consists of targeting the different ligands of TIM-3, notably Gal-9 recognized by α-lactose. Modulation of the TIM-3/Gal-9 checkpoint can be achieved with both α- and β-lactose. Moreover, lactose is a quasi-pan-galectin ligand, capable of modulating the functions of most of the 16 galectin molecules. The present review provides a complete analysis of the pharmaceutical and galectin-related biological functions of (α/β)-lactose. A focus is made on the capacity of lactose and Gal-9 to modulate both the TIM-3/Gal-9 and PD-1/PD-L1 immune checkpoints in oncology. Modulation of the TIM-3/Gal-9 checkpoint is a promising approach for the treatment of cancers and the role of lactose in this context is discussed. The review highlights the immuno-regulatory functions of lactose, and the benefit of the molecule well beyond its use as a pharmaceutical excipient.

## 1. Introduction

The modulation of cancer immunity represents the most potent therapeutic modality in oncology. Over the past ten years, the clinical approval of different immune checkpoint blockers has led to considerable improvement in the clinical outcome of cancer patients, notably those with solid tumors such as melanoma and lung cancer. However, for several other major cancers, death rates continue to increase, or the improvement progresses very slowly [1]. Research efforts must continue and novel approaches to improve prevention, early detection, and treatment are needed.

Novel immune checkpoint targets are proposed to offer new therapeutics and modalities to treat aggressive cancers such as pancreatic cancer, brain tumors, pleural mesothelioma, and triple-negative breast cancer for example [2,3,4,5,6,7]. Intense research efforts are also dedicated to improving the treatment of onco-hematological diseases. Targeting the immune system is considered a privileged axis to combat acute myeloid leukemia (AML) which is a highly aggressive disease, or to fight lymphoma in young patients, and multiple myeloma [8,9,10]. One of the emerging immune checkpoint molecules is the T-cell immunoglobulin mucin-3 (Tim-3)/Galectin-9 (Gal-9) checkpoint, viewed as a promising target for the treatment of breast cancer, bladder cancer and others [11,12,13,14]. Blockade of the TIM-3/Gal-9 checkpoint is considered a valid option in hematological malignancies, notably for the treatment of AML and myelodysplastic syndromes [15,16,17,18]. In the present review, we have analyzed the targeting of the TIM-3/Gal-9 checkpoint with various molecules, small and large, and analyzed specifically the potential benefit of lactose as an immune checkpoint regulator. The various biological functions of this extensively used natural molecule are underlined.

## 2. Lactose: α- and β-Anomers

**Figure 1:**
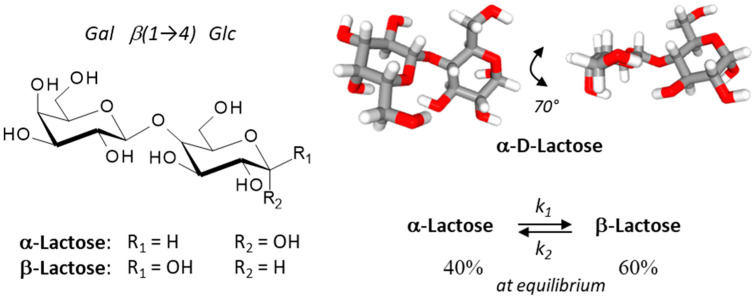
Structures of the two lactose anomers, α-lactose and β-lactose. Lactose contains a β-D-galactose unit linked to an α or a β-D-glucose unit through a β(1→4) bond. α-Lactose presents two crystalline forms, anhydrous α-lactose (C_12_H_22_O_11_) and α-lactose monohydrate (C_12_H_22_O_11_•H_2_O). The stability of lactose in aqueous solution has been determined [22]. An overall lactose epimerization rate constant (k) of 4.4 × 10^−4^ s^−1^ was measured at 25 °C (forward rate constant k_1_ of 2.5 × 10^−4^ s^−1^, and a reverse rate constant k_2_ of 1.5 ×10^−4^ s^−1^; K = 1.6 ± 0.1) and the half-life (t_1/2_) was 28.3 min at 25 °C. At equilibrium, the α/β anomeric ratio of lactose samples was about 39–41/59–61%, as determined at 25°, 45° and 60 °C.

Lactose is a disaccharide composed of a galactose unit linked to a glucose unit via a β-1,4 linkage (4-O-β-D-galactopyranosyl-D-glucopyranose, or Gal β(1→4) Glc). There are two anomers, α- and β-lactose, which differ in the configuration of the C-1 hydroxyl group of the glucose unit. α-Lactose (α-L) has an axial hydroxyl whereas the hydroxyl of β-lactose (β-L) is equatorial (Figure 1). The two anomers coexist in a solution, with an equilibrium of about 40% α-L and 60% β-L at room temperature. The α-anomer presents three crystalline forms: α-lactose monohydrate (α-LM), hygroscopic anhydrous α-lactose (α-LH) and stable anhydrous α-lactose (α-LS). The β-anomer (β-L) has one crystalline form [19,20]. The polymorphs of lactose exhibit distinct physical properties, in terms of solubility, density, stability, shelf life, dissolution rate, and bioavailability. α- and β-lactose differ considerably in solubility, β-L being much more water-soluble than α-L [21]. The interconversion between the different forms has been well investigated. During the mutarotation process, a short-lived hemiacetal intermediate is generated upon a ring-opening reaction of the glucose unit [22,23]. Similarly, the conversion of α-LM to the anhydrous forms is well-controlled [24]. The α/β lactose epimerization reaction occurs rapidly in aqueous solution (t_1/2_ = 28.3 min at 25 °C) and the product is stable [22]. An efficient method for the preparation of pure β-L from α-LM has been reported recently [25,26].

Lactose is used as an additive in many foods and as a filler or diluent in pharmaceutical products. α-LM is an excipient commonly used for the manufacturing of dry granules and tablets. However, β-L anhydrous is also used in direct compression of tablet processes and as a filler in capsules [27]. As an excipient, lactose is present in nearly half of all solid medicines because it is readily available, cost-effective, chemically stable, and easily compatible with other excipients and active ingredients and has a bland taste. It provides a convenient matrix to stabilize different types of active pharmaceutical ingredients (API). α-Lactose monohydrate is also one of the most used carriers for dry powder inhaler formulations [28,29,30]. The lactose used as an excipient has generally a very high anomeric purity (about 96% α and 4% β) although the anomeric content of α-LM can vary significantly from one batch to another, and this variation can influence bioavailability from final formulations [31]. In solution, a rapid lactose epimerization process can occur [22]. Dehydration leads to solid-state epimerization in α-lactose powders [32]. The form of lactose can be extremely important as it can serve to modulate the property of the active ingredient. For example, the use of highly porous lactose as a carrier can significantly improve the solubility of the flavonoid quercetin and its release, compared to a conventional *α*-lactose carrier [33]. The lactose excipient can influence the dissolution properties of the API present in the drug tablet, notably to improve the dissolution behavior of an insoluble API. Different types of lactose-based excipients and co-excipients are available [34,35].

Lactose is considered a pharmacologically inactive substance, which is the definition of an excipient. However, lactose is not devoid of biological activity. People intolerant to lactose may experience severe clinical symptoms (such as diarrhea and bloating); the disease severity varies considerably among individuals. The intolerance is generally due to the absence or deficiency of the lactase enzyme (lactase-phlorizin hydrolase) found in the small intestinal brush border, which hydrolyses lactose into glucose and galactose. Lactose intolerance is a relatively common gastrointestinal condition, at least in children, caused by the inability to digest and absorb dietary lactose [36,37]. Lactose can serve as an inducer of protein synthesis in cell cultures [38] and can induce senescence in human lung fibroblasts [39]. Moreover, in recent years the immunological functions of lactose have been delineated, notably its role as a galectin ligand and more specifically as a key regulator of the TIM-3 receptor. The present review highlights the importance of lactose as a checkpoint regulator.

## 3. The TIM-3 Immune Checkpoint and Its Targeting with Antibodies

Monoclonal antibodies (mAbs) targeting critical immune checkpoints, such as PD-1/PD-L1 and CTLA-4, have significantly ameliorated the treatment of advanced solid tumors, such as lung (NSCLC), skin and liver cancers. Immune checkpoint inhibitors targeting and blocking the interaction of certain cell surface proteins, such as PD-1 expressed on diverse immune cells or PD-L1 expressed on cancer cells, act as brakes on immune responses [40,41]. A handful of mAbs directed against PD-1 or PD-L1 have been approved for cancer treatment, including pembrolizumab, nivolumab, atezolizumab, camrelizumab and others. Often combined with chemo- or radiotherapy, these mAbs have provided significant survival benefits in cancer patients [42]. However, not all tumors are sufficiently sensitive to anti-PD-1/PD-L1 therapies and durable responses are not always achieved. Therefore, novel immunotherapeutic approaches are needed to combat advanced or relapsed cancers. As for PD-1, other immune checkpoints commonly associated with T cells such as LAG-3 and TIM-3 have been considered [43,44].

**Figure 2:**
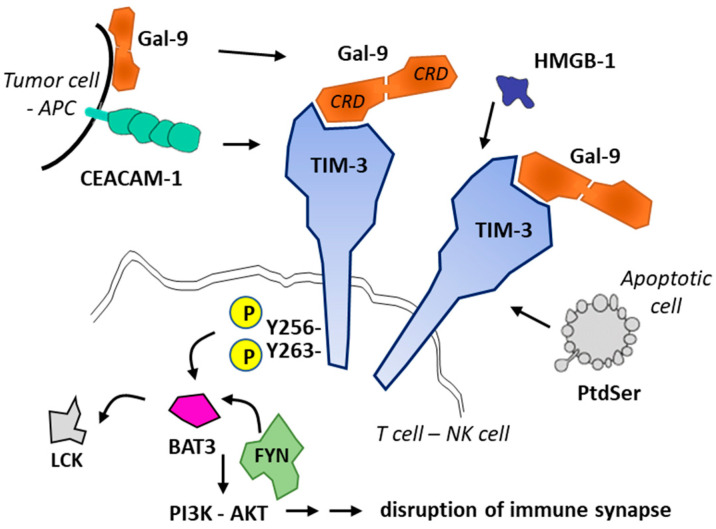
Ligands and signaling of TIM-3. Four TIM-3 ligands have been identified: CEACAM1, phosphatidylserine (PtdSer released from apoptotic cells), HMGB-1 and Gal-9. Gal-9 can be secreted by antigen-presenting cells (APCs) or tumor cells or present at the cell surface. Gal-9 (with two carbohydrate recognition domains (CRD) separated by a short linker) promotes oligomerization of TIM3 and triggers signaling via phosphorylation of residues Tyr256 and Tyr263 in the intracellular domain of TIM3. The phosphorylation releases the adaptor protein BAT3 (HLA-B-associated transcript 3) and allows recruitment of tyrosine kinase FYN, whereas in its ligand-unbound form, TIM-3 interacts with BAT3 and recruits the kinase LCK to maintain T cell activation. Fyn and Bat3 are two adaptor molecules involved in inhibition and activation of Tim-3 downstream signaling, respectively. Gal-9-mediated recruitment of FYN leads to the disruption of immune synapse formation and to cell apoptosis [45].

T cell immunoglobulin and mucin domain molecule 3 (TIM-3) is a protein now well exploited as a therapeutic target for cancer immunotherapy [45,46,47,48]. This co-inhibitory receptor is expressed on CD4^+^ and CD8^+^ T cells which produce interferon-γ (IFN-γ) and on specific innate immune cells (Figure 2). In addition, the expression of membrane TIM-3 has been evidenced at the surface of cancer cells, such as renal, lung, gastric, prostate, and other types of cancer cells. TIM-3 plays major roles in the regulation of tumorigenesis, inflammation, and antitumor immunity [49]. Importantly, TIM-3 is upregulated on CD8^+^ T cells and Tregs, in tumors treated with radiotherapy and anti-PD-L1 antibodies. The treatment with an anti-TIM-3 mAb concurrently with anti-PD-L1 and radiotherapy induced a significant tumor growth delay, an enhancement of T-cell cytotoxicity, a decrease of Tregs, and improved survival in models of head and neck squamous cell carcinoma [50]. The data suggested that the blockade of Tim-3 can antagonize the resistance of PD-1/PD-L1 blocking therapy [51]. Major anticancer effects have been reported in cases of Tim-3 blockade, in different types of experimental tumors, incusing breast cancer [12,52], hepatocellular carcinoma [53], and other solid tumors [54]. The blockade of TIM-3 is also a promising approach for the treatment of hematological malignancies, including myelodysplastic syndromes (MDS) and acute myeloid leukemia (AML) [17,55,56,57]. In AML, studies have shown an overexpression of TIM-3 on leukemia stem cells (LSC) but not on healthy stem cells [58,59,60]. The blockade of the receptor induces a direct inhibition of AML cell proliferation and restores T cell function [16]. Thus, targeting TIM-3 offers a unique opportunity to target the LSC population resistant to chemotherapy and largely implicated in tumor relapse [61].

**Figure 3:**
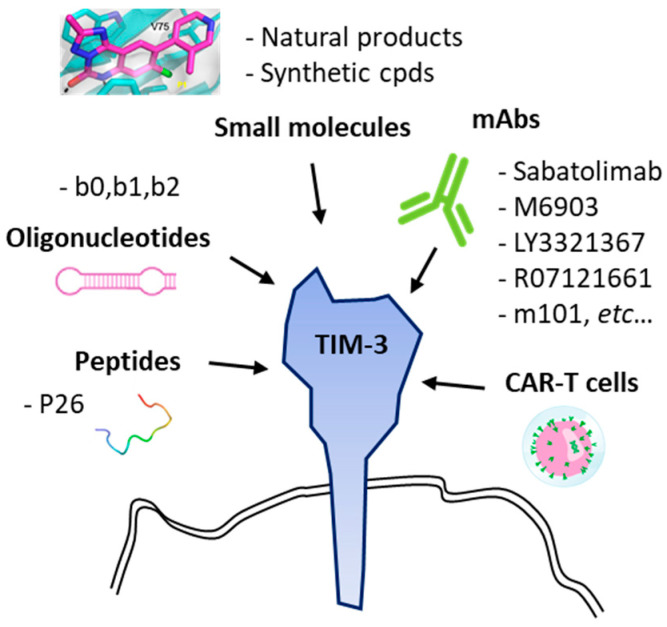
Targeting of TIM-3 with monoclonal antibodies (mAbs), oligonucleotides (such as nuclease-resistant 2’-fluoropyrimidine RNA aptamers [69]), peptides (12-mer P26 [71]) and small heterocyclic molecules (natural products and synthetic compounds).

There are several anti-TIM-3 monoclonal antibodies in (pre)clinical development [62], such as (i) sabatolimab which is currently evaluated for the treatment of advanced solid tumors [63] and myelodysplastic syndromes [64], (ii) the fully human anti-TIM-3 antibody M6903 in preclinical development [65], (iii) LY3321367 currently tested in advanced solid tumors [66], and a few other antibodies [49,67] (Table 1). Over the past two years, TIM-3 has emerged as an important immune-checkpoint molecule and recent data demonstrated that the receptor plays multiple roles, well beyond CD4^+^/CD8^+^ T cells, notably as a key regulator of dendritic cell functions via the regulation of inflammasome activation [68]. The discovery of the many functions of the TIM-3 receptor has encouraged the design and development of different therapeutic entities, monoclonal antibodies but also anti-TIM-3 chimeric antigen receptor (CAR)-directed T lymphocyte therapy [44] and anti-TIM-3 oligonucleotide aptamers [69,70] (Figure 3).

Anti-TIM-3 biotherapeutic agents have demonstrated potent efficacy in preclinical models and the first clinical data obtained with sabatolimab look promising for the treatment of MDS [78]. A phase 2 trial is ongoing in MDS with sabatolimab combined with azacitidine and venetoclax (NCT04812548). Combined with azacytidine, the anti-TIM-3 antibody might turn to be also useful for the treatment refractory acute myeloid leukemia (AML). A major phase 1–2 trial is currently ongoing (NCT04623216). However, the challenge remains high at present, as no definite proof of long-term clinical efficacy has been reported in AML or MDS. In CD8^+^ T cells collected from patients with chronic lymphocytic leukemia (CLL), the blockade of an anti-TIM-3 antibody has failed to restore the function of these exhausted T cells [79]. Moreover, a study has shown that the treatment with anti-TIM-3 mAb can inhibit the phagocytic ability of alveolar macrophages in the lungs of mice and thus might cause pneumonitis [80]. The biological and clinical consequences of TIM-3 blockade are not completely understood at present.

Therapeutic antibodies and engineered cells targeting TIM-3 have been designed and are being tested. However, to our knowledge, no small molecules specifically targeting TIM-3 have been clinically developed as yet, despite the potential benefits of the targeting approach in oncology and in other therapeutic fields [14]. The TIM-3-associated immune dysregulation mechanisms of CD4^+^ or CD8^+^ T cells have been associated with different inflammatory diseases such as vitiligo [81,82], rheumatoid arthritis [83,84], liver diseases [85] and in viral infections such as human papilloma virus (HPV) positive cervical cancer [86]. There is a need for small molecules targeting TIM-3, to reduce the cost of treatment compared to mAbs, to facilitate drug combinations and to allow oral treatments. Thus far, the development of small molecules lags far behind the development of small molecules, as discussed below.

## 4. Small Molecules Targeting TIM-3/Gal-9 Checkpoint

Clinical developments have been initiated with different types of monoclonal antibodies targeting TIM-3 or co-targeting TIM-3 and PD-1 with bispecific antibodies, such as the mAb R07121661 from Roche (Phase 1 clinical trial NCT03708328 in advanced cancers). Beyond mAbs, TIM-3 has been targeted with a few peptides and oligonucleotides. Peptides targeting TIM-3 can provide anticancer effects in vitro and in vivo, such as the 12-mer peptide P26 (GLIPLTTMHIGK) which binds to TIM-3 with a relatively modest affinity (K_D_ = 3.44 × 10^−5^ M). Peptide P26 blocks the interaction between TIM-3 and its ligand galectin-9 (Gal-9) so as to reverse Gal-9-inhibited T cell activation and to trigger T-cell and cytokines-dependent anticancer effects in an MC38 model of murine colon cancer [71]. Alternatively, TIM-3 can be targeted with aptamer oligonucleotides, such as a trimeric 20-fluoropyrimidine RNA oligonucleotide which has been shown to bind to TIM-3 and to enhance T-cell functions leading to a marked potentiation of tumor immunity in mice [69]. However, apart from these two examples, the targeting of TIM-3 with non-antibody molecules has proven difficult. In general, the targeting of immune checkpoints with small molecules is a major challenge, rarely crowned with success [87].

**Figure 4:**
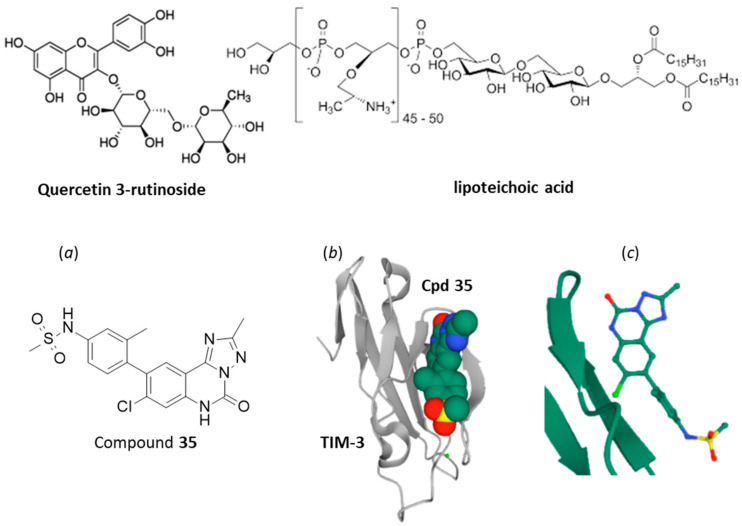
Small molecules known to bind to TIM-3 (or to regulate expression). (**top**) Rutin and lipoteichoic acid. (**bottom**) (**a**) Structure of the triazoloquinazolinone derivative 35. (**b**) Molecular model of cpd 35 bound to TIM-3. (**c**) A detailed view of Cpd 35 interfaced with the protein [95].

In recent years, a few approaches are emerging to identify small molecules directed against TIM-3. First, a mass spectrometry-based screening method has led to the identification of a few hit compounds (designated NP-434, NP-592 and NP-610) able to bind to TIM-3. They were identified as being flavanone/flavone glycosides, such as compound quercetin 3-O-rutinoside (NP-610 or rutin, Figure 4) which can form stable complexes with TIM-3 [88]. This flavonoid found in diverse plants is interesting because it has revealed anticancer effects in vitro [89,90]. Second, the natural product lipoteichoic acid (a cell wall component of Gram-positive bacteria, such as Bifidobacterium) has been shown to inhibit the TIM-3/Gal-9 pathway and to reduce the immunosuppressive activity of CD4^+^ CD25^+^-Treg to enhance cell-mediated immunity [91]. The compound down-regulated expression of TIM-3 and significantly enhanced the antitumor effect of 5-fluorouracil in Hepatoma-22 tumor-bearing mice [92]. Lipoteichoic acid (LTA, Figure 4) is a glycosylated anticancer natural product, active against different types of cancer cells (e.g., gastric cancer [93], but it is also a chemically complex and toxic molecule (used experimentally to induce acute lung injury [94]). Therefore, it is not a convenient model for drug design. Third and more interestingly, small molecule fragments capable of interacting with TIM-3 have been recently identified through an NMR-based fragment screen and optimized. Starting from low-affinity fragments such as the tricyclic triazoloquinazolinone unit (K_D_ = 810 µM), the authors designed and synthesized high-affinity TIM-3 small-molecule ligands, such as compound the sulfonamide derivative 35 (Figure 4) with a strongly reinforced affinity for TIM-3 (K_i_ = 156 nM) [95]. This is a clever and promising approach toward the discovery of TIM-3-targeted therapeutics, even if at present the anticancer potential of these molecules has not been reported. This study is important as it demonstrates that TIM-3 can be targeted with small molecules.

Another approach to tackle TIM-3 would consist of neutralizing its ligands, not binding directly to the receptors, or blocking the downstream signaling pathway. The human Tim-3 protein includes 301-amino acids, with an extracellular region containing a Cys-rich Ig-like domain and a Ser/Thr-rich mucin domain. The C-terminal extremity oriented toward the cytosol mediates intracellular signaling [96]. Several ligands for TIM-3 have been identified: galectin-9 (Gal-9), phosphatidylserine (PtdSer), high-mobility group protein B1 (HMGB1), and the carcinoembryonic antigen-related cell adhesion molecule-1 (CEACAM-1) (Figure 2) [97]. Each ligand provides opportunities to interfere with the correct functioning of the receptor. For example, it has been shown that TIM-3 inhibits the antitumor efficacy of DNA vaccines and chemotherapy by binding to HMGB1 [6], suggesting therefore that the neutralization of HMGB1 can be an option to reduce the effect of TIM-3. There are small molecules, such as glycyrrhizin [98,99], to target HMGB1 [100,101]. Another option is targeting Gal-9, which belongs to the family of β-galactoside-binding lectins and offers a binding site for lactose. The approach is described below.

## 5. Tim-3/Gal-9 Signaling Blockade with α/β-Lactose

Galectins (Gal) are carbohydrate-binding proteins with a high affinity for β-galactosides present in glycoproteins and glycolipids. They play important roles in the immune response via the regulation of homeostasis and immune functions. They are essential in the regulation of virus infections [102]. There are sixteen types of galectins and several of them, such as Gal-1, -3, -9 and -12 have been implicated in the development and progression of leukemia [103]. Gal-9 (also known as ecalectin) is a 36 kDa β-D-galactoside mammalian C-type lectin [104,105]. Gal-9 was the first ligand identified for TIM-3, interacting via the carbohydrate moiety of the protein [106].

**Figure 5:**
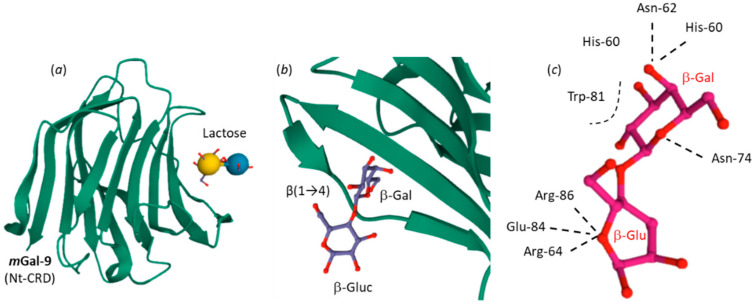
Crystal structure of mouse Gal-9 N-terminal CRD domain (NCRD) bound to lactose (PDB: 2D6M). (**a**) Lactose bound to mGal-9. (**b**) A close-up view of bound lactose. The β-galactoside moiety is deeply buried in the binding site formed by the juxtaposed β-strands. (**c**) Conformation of the bound lactose molecule and H-bonds formed with specific amino acid residues of mGal-9 [107].

The crystal structure of lactose bound to the N-terminal carbohydrate recognition domain (CRD) of mouse Gal-9 has been solved [107]. The β-galactoside moiety is inserted into the protein, in contact with three juxtaposed β-strands. Several H-bond interactions stabilize the complex, as represented in Figure 5. O4 of the galactose moiety in contact with His-60, Asn-62 and Arg-64 residues, and O6 of the glucose moiety in contact with Arg-64, Glu-84, and Arg-86 residues are essential to the interaction. The Arg-64 residue is key because its replacement with Ala in mouse Gal-9 impairs its capacity to bind to TIM-3 [106]. Stacking interactions with His-60 and Trp-81 also contribute to the stability of the lactose-Gal-9 complex.

**Figure 6:**
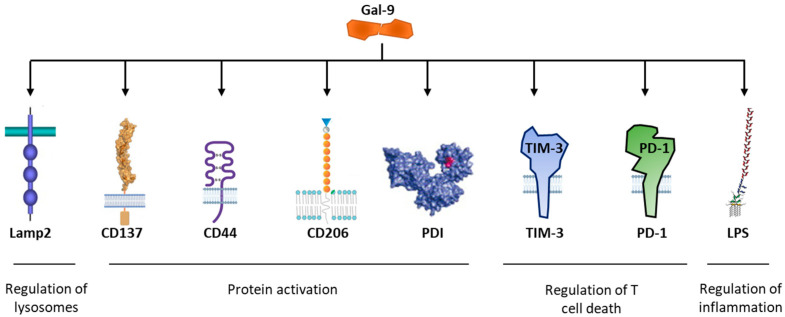
Gal-9 interacting partners: C137 (also known as 4-1BB) belonging to the TNF receptor superfamily; the CD44 adhesion molecule; the macrophage M2 biomarker CD206; protein disulfide isomerase (PDI) via binding to its *O*-glycans; the lysosomal associated membrane protein 2 (Lamp2); bacterial lipopolysaccharide (LPS) and the checkpoints TIM-3 and PD-1. The list is non-exhaustive. As a glycan-binding immunomodulatory factor, Gal-9 can have numerous glycosylated protein partners.

The binding of Gal-9 to the extracellular domain of TIM-3 regulates the fate of T cells, notably via the phosphorylation of the conserved tyrosine Y265 residue in the intracellular tail of the protein [108] (Figure 2). Gal-9 has two peptide-linked carbohydrate recognition domains (CRDs), but alternative splicing and proteolytic processing can generate several isoforms [109,110]. Gal-9 has several receptors (Figure 6): the CD44 adhesion molecule [111], the macrophage marker CD206 (mannose receptor) [112], the lysosomal associated membrane protein 2 (LAMP2) [113], the TNF receptor superfamily member CD137 (also known as 4-1BB) [114], protein disulfide isomerase (PDI) implicated in T cells migration [115,116], the two key immune receptors PD-1 and TIM-3 [117], bacterial lipopolysaccharides and other proteins equipped with N-glycans [109,118]. Gal-9 has multiple biological functions in cell growth, differentiation, adhesion, communication and death, and is a marker of disease severity [119]. The protein, expressed on macrophages and tumor cells, is considered as an important target in oncology because, on the one hand, high Gal-9 expression correlates with poor prognosis in multiple human cancers and, on the other hand, an anti-Gal-9 antibody has been shown to selectively expand intratumoral TIM-3^+^ cytotoxic CD8 T cells and immunosuppressive regulatory T cells [117]. Gal-9 plays a role in both innate immunity and acquired immunity via the induction of maturation of dendritic cells [120]. It is a potent inducer of cancer cell death in lymphomas and other malignant cell types, such as prostate cancer cells [121] and colon cancer [122]. However, Gal-9 is a complex and fragile molecule, with a largely expressed plasma membrane form [123], an intracellular form [124] and a circulating soluble form [125]. The expression and secretion of Gal-9 is promoted by interferon β and γ [126].

An elevated level of Gal-9 has been measured in the serum of AML patients and in mice xenografted with primary human AML. In parallel, the treatment with an anti-Gal-9 antibody was found to prevent AML development in mice, via the targeting of the leukemia stem cells population [58,59,60]. The data indicated that, via an interaction with TIM-3, Gal-9 exerts significant immunomodulatory effects by inducing apoptosis or suppressing effector functions. Blocking Gal-9 signals to TIM-3-expressing T cells improved the immune response. These effects can be highly profitable to combat cancers and viral diseases [127].

Lactose, in the form of β-lactose, is considered as a pan-galectin inhibitor, capable of binding to almost all galectin proteins, with a few exceptions (Table 2). For example, lactose does not bind Gal-16 which lacks the ability to bind the β-galactoside lactose [128]. β-L is a pan-galectin ligand that can be used as a model to elaborate biomimetic to modulate the activity of lectins. For example, cyclodextrins, cyclic peptides, calixarenes and glycophanes have been designed from β-L [129]. Various lactosides and glycomimetics can be used to design high affinity and selective galectin antagonists, especially for human Gal-1 and Gal-3 which are largely studied [130,131,132,133,134,135]. Gal-3 is an attractive anticancer target, notably for breast and endometrial cancers [136,137,138]. The targeting of Gal-9 has been less investigated at present, at least in terms of small molecules targeting, although it is considered an excellent anticancer target [139]. Human recombinant galectin-9 proteins have demonstrated robust anti-cancer activities in different models, such as in cutaneous T cell lymphoma (CTCL) [140] and in a model of chronic myelogenous leukemia [141]. Gal-9 enhances tumor immunity mediated by T cells, macrophages, and dendritic cells. A stable, proteolysis-resistant, mutant form of Gal-9 has been shown to display marked anticancer effects in a murine model (KYSE-150) of esophageal squamous cell carcinoma (ESCC) [142].

The first demonstration of the capacity of α-lactose to modulate the Gal-9/TIM-3 interaction was reported in 2005. It was shown that α-L could function as a competitive substrate to dose-dependently inhibit the interaction between recombinant Gal-9 bound to both full-length and soluble TIM-3–Ig fusion proteins in solution [106]. Later, Sehrawat and coworkers (2010) showed that α-L reduced Gal-9 binding to TIM-3 and reversed Gal-9 inhibition of CD8^+^ T cell response in a model of Herpes Simplex Virus (HSV) infection [162]. Subsequently, the neutralization of Gal-9 with α-L was observed in epithelial cells, preventing the secretion of IFN-γ [163]. α-L has also been used to modulate the Gal-9/TIM-3 signaling pathway and to modulate the M1/M2 macrophage subtype polarization balance in mice [164,165]. Similarly, α-L was used to block Gal-9/TIM-3 interaction in macrophages in order to attenuate the bactericidal activity of intracellular *Salmonella typhimurium* [166]. High concentrations of α-L (25–100 mM) are generally required to modulate the Gal-9/TIM-3 interaction in vivo [166,167]. The use of α-L provides a convenient means to decrease immune suppression via the modulation of the Gal-9/TIM-3 interaction in a model of chronic stress-induced inflammation, with reduction of the autophagy level [168] and in lymphocyte populations isolated from mice infected with the malaria parasite *Plasmodium berghei* [169,170]. α-L has been used in other studies as a blocker of Gal-9 binding to TIM-3 [171,172], notably in the frame of studies on sepsis. The blockade of TIM-3 signaling with α-L was found to prevent apoptosis of NK T cells, to attenuate the production of inflammatory cytokines (such as IL-12) and to improve markedly the survival of septic mice (upon hypodermic injection of α-L 5–10% solution) [173]. Similarly, α-L was found to reduce liver inflammation in an experimental model of sepsis in mice (cecal ligation and puncture model), suppressing TIM-3 expression in liver CD8^+^ T cells [174]. The capacity of lactose to modulate cellular activities via Gal-9 binding has been evidenced in different cell and animal models (Table 3).

However, is the capacity of lactose to antagonize Gal-9 binding to TIM-3 specific for the α anomer? Although there is no published comparison of the effect of the two α/β anomers, the answer to the question is probably no. In a study, the blockade of the association of Gal-9 to Tim-3 on the cell surface of osteoclast precursors was achieved with β-L [179]. In addition, β-L has been mentioned in a publication (in Chinese) for its capacity to inhibit the Gal-9 mediated regulation of E-selectin expression in colon carcinoma LoVo cells [180]. Other studies used β-L to regulate Gal-9 expression and function in cells [181,182]. Importantly, a study has demonstrated that β-L (30 mM) abrogated IFN-γ production within Tim-3–expressing NK-cell populations [183]. Therefore, for these reasons we suspect that the capacity of TIM-3 to modulate the Gal-9/TIM-3 checkpoint is not specific to α-L but can occur with both α-L and β-L. This would also be logical, because the C-1 OH group of the glucose moiety of lactose is not directly engaged in the interaction with the CRD of Gal-9 (Figure 5). As mentioned above, in solution the two anomeric forms of the disaccharide (α/β) easily undergo mutarotation and interconversion to reach dynamic equilibrium. Most TIM-3 related studies have been performed with α-L, but laboratory solutions likely contain a mixture of the α and β anomers. As for other disaccharides, the forward α→β anomeric interconversion of lactose is facilitated compared to the backward β→α anomer interconversion. Recently, under specific experimental conditions (20 °C, 2 mM ammonium acetate) t_1/2_ of 8.2 and 12.2 min have been calculated for forward (α→β) and backward (β→α) interconversion of lactose [184]. It takes about 4 h for a solution of α-L to reach the equilibrium content of 38% α-L and 62% β-L at 25 °C [22].

## 6. Lactose as a Dual Modulator of Gal-9/TIM-3 and PD1/PD-L1 Checkpoints

**Figure 7:**
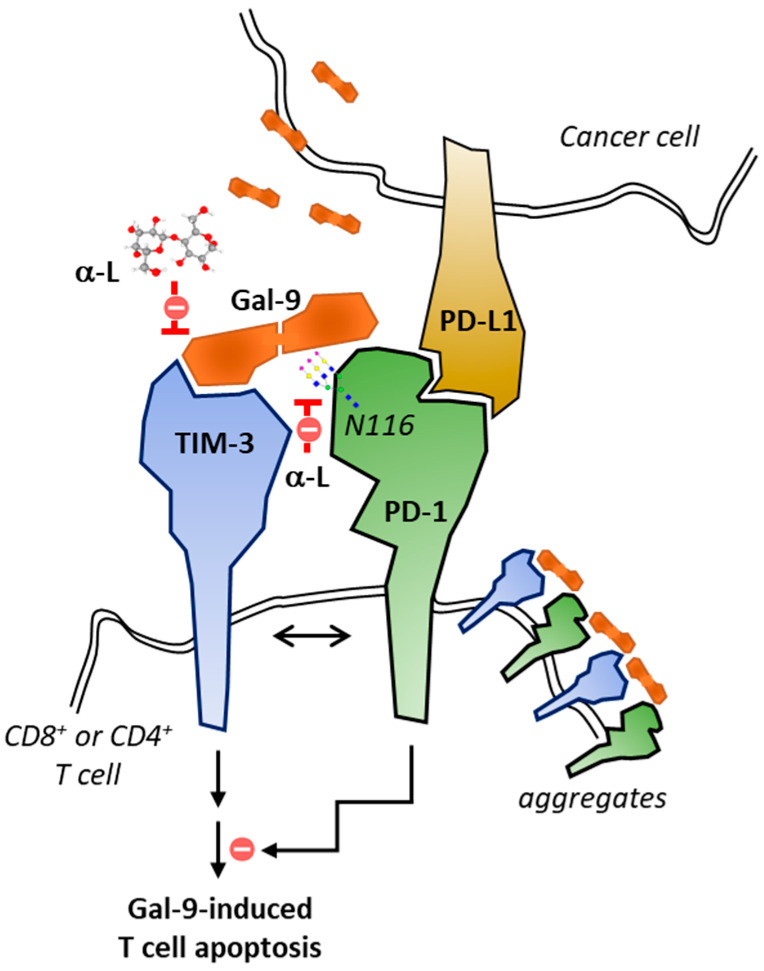
The interplay between PD-1 and TIM-3 and the dual function of Gal-9. Gal-9 is frequently expressed on cancer cells and exists as a soluble protein. Gal-9 has been shown to bind both TIM-3 and PD-1, and to induce apoptosis of CD4^+^ or CD8^+^ T cells. However, the co-expression of PD-1 protects TIM-3^+^ T cells from Gal-9-induced cell death. Gal-9-binding takes place via the N116-linked glycan of PD-1. Gal-9 triggers the formation of TIM-3/PD-1 aggregates. The interaction can be modulated with anti-Gal-9 therapies (adapted from [117]).

Lactose is remarkable because it can suppress the interaction of Gal-9 with the two T cell inhibitory receptors PD-1 and TIM-3 which are co-expressed on T cells. Indeed, recently Yang and co-workers made the landmark discovery that Gal-9 can bind PD-1 receptor and the interaction can be interrupted by lactose (but not sucrose) [117]. Apparently, Gal-9 binds to a site on PD-1 distinct from the PD-L1-binding site because PD-L1 binding is not affected in presence of Gal-9 (Figure 7). Binding seems to occur through an N-glycan of PD-1 (at asparagine N116) recognized by the C-terminal carbohydrate-recognition domain (CRD) of Gal-9, as represented in Figure 6. As such, TIM-3 and PD-1 would form heterodimers via their juxtaposed intracellular domains and these structures can be bridged with Gal-9 to form galectin-glycoprotein lattices. The crosstalk between the two receptors and the functional role of the resulting aggregates warrant further investigation but the authors suggested that co-expressed PD-1 suppresses Gal-9/TIM-3-induced T cell apoptosis and favors the proliferation and differentiation of these cells (feedback mechanism) [117]. These key observations, together with the demonstration of a marked anticancer activity with anti-Gal-9 antibodies, make Gal-9 a very attractive target for the treatment of cancers. The control of Gal-9 expression and secretion emerges as a promising anticancer strategy. The use of Gal-9 ligands, including lactose, could be useful to modulate the T cell inhibitory receptors PD-1 and TIM-3. No doubt that novel anti-Gal-9-based therapies will be proposed soon. The membranous expression of Gal-9 is frequent in tumors [185,186]. For example, it has been observed in 82% of squamous cell carcinomas, and the dual TIM-3/Gal-9 expression was observed in 86% of PD-L1-positive cases and 100% of PD-L1-positive squamous cell carcinomas [187]. In onco-hematology, Gal-9 and PD-L1 were found to be both upregulated in patients with chronic lymphocytic leukemia [188] and Gal-9/Tim-3 expression levels were found higher in AML patients who fail chemotherapy [57]. A concerted approach to target simultaneously the two checkpoints would be logical. In this context, lactose or derivatives, or functionally equivalent products could be useful.

## 7. Conclusions and Perspectives

In recent years, the checkpoint receptor TIM-3 has emerged as an essential molecule in the pathogenesis of different forms of cancer, viral diseases and other pathologies. This receptor contributes importantly to the dysfunctional activity and exhaustion of T cells. The blockade of TIM-3 results in tumor regression in preclinical models and the first clinical data obtained with anti-TIM-3 antibodies are very encouraging, at least in onco-hematology (for MDS and AML in particular). The co-blockade of the two checkpoints TIM-3 and PD-1 look very promising for advanced cancers [49,62,63,70]. TIM-3 seems to play a significant role in a compensatory inhibitory mechanism which develops upon treatment with anti-PD-1 and PD-L1 antibodies in breast cancer [189,190]. In leukemia, TIM-3 is an essential receptor for the self-renewal of leukemic stem cells and a great target for anti-leukemia therapy [191]. Clearly, anti-TIM-3 antibodies hold great promise in oncology.

Beyond mAbs, TIM-3 can be targeted with smaller molecules, such as peptides and RNA aptamers, and with chemical compounds, even if the design of small molecule inhibitors has proved challenging. Recently, the lock has been released. Rietz and coworkers have demonstrated that it is possible to directly target TIM-3 with specifically designed small heterocyclic molecules presenting nanomolar affinities of the receptor [95]. Alternatively, the TIM-3 checkpoint can be regulated with molecules that binds to the Gal-9 ligand, such as the disaccharide lactose. α-Lactose (α-L) functions as a Gal-9 binder able to regulate TIM-3 function and expression in cells. The compound is active in vitro and in vivo, and devoid of toxic effects. It is one of the very classical excipients used in drug manufacturing and a useful tool to study the biology of the TIM-3 checkpoint and its regulation. However, a proper knowledge and considerations of the specific chemical and pharmaceutical properties of α-L are mandatory. In a few cases, modulation of the TIM-3/Gal-9 checkpoint has been reported with β-L [183]. Therefore, we consider that both anomers may function as regulators of TIM-3. A proper comparison of the Gal-9-binding capacity of α-L and β-L is required, and an analysis of their TIM-3-modulating effects. The comparison is important and necessary, notably because of the extended use of α-L as a drug excipient in many solid drug formulations.

The binding of lactose (at least α-L) to Gal-9 raises opportunities to extend studies to other related lactose derivatives, such as the tumor and pathogenic glycol-biomarker LacdiNAc (GalNAcβ1-4GlcNAc) and the ubiquitous LacNAc epitope (Galβ1-4GlcNAc), both known to bind to Gal-3 [192] and to mouse Gal-9 [107]. Gal-9 recognizes many galactoside-containing carbohydrates, including the Thomsen-Friedenreich antigen (T-antigen, Gal1-3GalNAc) [107]. It will be interesting to see if these lactose derivatives can also regulate TIM-3 expression and function in cells. More openly, investigations of anti-TIM-3 activity could be considered with other lactose-containing derivatives, such as the myricetin M10, a natural dietary flavonoid equipped with a 3-O-β-D-lactose unit [193]. M10 has been shown to prevent infiltration of myeloid-derived suppressor cells and increase CD8^+^ T and CD4^+^ T cells in colorectal tissues [194]. The lactose unit of this potent anti-inflammatory compound, considered for the treatment of ulcerative colitis [195,196], may contribute to a modulation of the TIM-3/Gal-9 checkpoint. The recognition of Gal-9 by lactose makes this natural product an interesting synthon for the design of glycodrugs. However, there is a caveat: lactose is not a selective Gal-9 binder but a quasi-pan-galectin ligand. Both the facile α/β anomeric conversion of lactose and the non-selective binding to galectins need to be considered in immuno-pharmacology studies of lactose.

Finally, we must conclude with cautionary notes regarding TIM-3 as a target and lactose as a modulator. Encouraging response rates have been reported with anti-TIM-3 antibodies for MDS and AML treatments, but the effect of TIM-3 blockade has been much less promising in patients with chronic lymphocytic leukemia [16]. TIM-3 is no cure-all for every cancer, and we need to comprehend further the biology of the target [197]. The use of lactose as a modulator of TIM-3/Gal-9 is interesting but it raises questions because it is a very common molecule in food and a common pharmaceutical excipient. We need to clarify the potential effects of lactose on the checkpoint and the possible interference with anti-TIM-3 antibodies.

## Figures and Tables

**Table 1:** Human anti-TIM-3 antibodies in development.

Anti-TIM-3 Antibodies	Current Development	References
LY3321367 (Eli Lilly; Indianapolis, IN, USA)	Phase 1 trial in patients with relapsed/refractory solid tumor, alone or in combination with an anti-PD-L1 mAb. NCT03099109 *	[66,72]
Sabatolimab (MBG453, Novartis, Basel, Switzerland)	Fast track designation by US FDA for the treatment of patients with myelodysplastic syndromes (MDS)	[73]
	Phase 1 trial in patients with advanced solid tumor, alone or in combination with anti-PD-1 spartalizumab.	[63]
	Phase 1 trial in patients with Acute Myeloid Leukemia (AML). NCT04812548, NCT04623216, NCT04878432, NCT04150029 *	[74]
BMS-986258 (ONO 7807, BMS, Lawrenceville, NJ, USA)	Fully human anti-TIM-3 mAb, tested in combination with anti-PD-1 nivolumab in patients with advanced solid tumors (Phase 1). NCT03446040 *	
Cobolimab (TSR-022, GSK, Brentford, UK)	Anti-TIM-3 mAb in combination with anti-PD-1 in patients with liver cancer or with a melanoma (Phase 1). NCT04655976, NCT03680508, NCT04139902, NCT02817633 *	
Sym023 (Symphogen; Ballerup, Denmark)	Fully human anti-TIM-3 mAb, in patients with advanced solid tumor malignancies or lymphomas (Phase 1). NCT03489343 *	
INCAGN02390 (Incyte, Agenus; Wilmington, DE, USA)	Phase 1 study to determine the safety, tolerability, and preliminary efficacy in participants with advanced malignancies. Fully human Fc-engineered IgG1k. NCT03652077, NCT04370704.	[75]
RO7121661 (Roche; Basel, Switzerland)	Anti-PD-1/TIM-3 bispecific mAbs, tested in patients with advanced and/or metastatic solid tumors (Phase 1). NCT03708328 *	
BGB-A425 (BeiGene Ltd., Beijing, China)	Humanized anti-TIM-3 mAb, in combination with anti-PD-1 mAb tislelizumab, in patients with advanced solid tumors (phase I/II trial). NCT03744468 *	[76]
M6903	Fully human anti-TIM-3 mAb, without effector function, which blocks binding of TIM-3 to the 3 ligands phosphatidylserine, CEACAM1, and Gal-9. Experimental laboratories studies.	[65]
F38.2E2	Anti-human TIM-3 antibody capable of blocking binding of TIM-3 to phosphatidylserine and CEACAM1. Experimental tool.	[77]

* ClinicalTrials.gov Identifier.

**Table 2:** Lactose binding to galectins.

Galectins	Lactose Binding	+/−	References
Gal-1	Lactose binds the two carbohydrate recognition domains of the Gal-1 dimer.	+	[143]
Gal-2	Lactose binds to only one of the carbohydrate recognition domain subunits of the Gal-2 dimer structure.	+	[144]
Gal-3	Crystal structure of the carbohydrate recognition domain of Gal-3 in complex with lactose.	+	[145,146]
Gal-4	Analysis of lactose and derivatives binding to C-terminal carbohydrate recognition domain of human Gal-4.	+	[147,148]
Gal-5	A little-studied galectin (apparently specific for rat). Lactose binding described.	+	[149,150]
Gal-6	Lactose binding not reported but very likely considering the strong homology with Gal-4.	?	[151,152]
Gal-7	Lactose binding induces stabilization of the Gal-7 dimer.	+	[153]
Gal-8	Binding of lactose to human galectin-8-N-domain	+	[154]
Gal-9	Structure of murine Gal-9 n-ter CRD bound to lactose.	+	[107]
Gal-10	Gal-10 forms a novel dimeric structure and binds lactose.	+	[155]
Gal-11	Gal-11 is only expressed in ruminants. Binding to lactose suggested.	+	[156]
Gal-12	Binding of lactose to Gal-12	+	[157]
Gal-13	Wild-type Gal-13 and its variant R53H do not bind lactose. Engineering of variant R53H can lead to lactose binding.	−	[158,159]
Gal-14	Lactose does not interact with this lectin, or very weakly.	−	[160]
Gal-15	Binding of lactose to Gal-15	+	[161]
Gal-16	Gal-16 exists as a monomer and lacks the ability to bind lactose.	−	[128]

**Table 3:** Lactose binding to galectin-9 in different biological systems.

Cell System or Animal Model	Effect of Gal-9	References
Endometrial regenerative cells (ERC)	ERC express Gal-9 and play a major role in immune modulation. Lactose blocks Gal-9 immunomodulatory effect in ERC, and thereby modulate the proliferative rate of stimulated CD4^+^ T and CD8^+^ T cells, cocultured with ERC.	[175]
Mice infected with the malaria pathogen *Plasmodium berghei*	Blockade of Tim-3/Gal-9 with α-lactose induces a compensatory expression of the immunosuppressive molecule TIGIT.	[170]
Bone marrow derived macrophages (BMDM).	Downregulation of Gal-9 and TIM-3 protein expression and soluble Gal-9 secretion in LPS-induced BMDM.	[165]
Prostate cancer cells (PC-3)	Addition of lactose induces solubilization of membrane-bound Gal-9.	[121]
*Salmonella enterica* intestinal infection mouse model	Blocking Tim-3/Gal-9 interaction with α-lactose attenuates the bactericidal activity of intracellular *S. typhimurium* by macrophages.	[166]
Pleural fluid cells (PFC)	Gal-9 stimulates interferon-γ synthesis in PFC and lactose inhibits this effect.	[176]
Intestinal epithelial cells (IEC) and mouse model.	Lactose binding to Gal-9 inhibits the anti-allergy properties of the sulfated polysaccharide F-fucoidan from *Saccharina japonica*.	[177]
Co-cultures of human peripheral blood mononuclear cell (PBMC)-derived Treg and effector T cells (Teff).	Lactose inhibits the down-regulation induced by Treg of the secretion of IFN-γ and IL-17 in PBMC-Teff co-cultures. Lactose inhibits human Treg-mediated suppression of Th1 and Th17 immune responses.	[178]
Intestinal epithelial cells (IEC)	Neutralization of Gal-9 with lactose prevents the induction of IFN-γ secretion and suppresses the production of IL-10 by PBMC.	[163]
